# Detección precoz e intervención activa en el deterioro cognitivo: Un reto sanitario

**DOI:** 10.23938/ASSN.1178

**Published:** 2026-08-27

**Authors:** David Brugos Miranda, Rosa Larumbe Ilundáin

**Affiliations:** 1 Servicio Navarro de Salud-Osasunbidea Gerencia de Salud Mental de Navarra Área de Recursos Intermedios Pamplona España; 2 Servicio Navarro de Salud-Osasunbidea Hospital Universitario de Navarra Servicio de Neurología Pamplona España

Los cambios biomédicos y tecnológicos, junto con la extensión de la educación, los hábitos saludables y los sistemas de protección -como las pensiones y la atención socio-sanitaria- han permitido un aumento notable de la esperanza de vida. Este incremento en longevidad supone un enorme desafío para el individuo que envejece, para su familia, y para la sociedad.

La edad trae consigo cierto declinar en algunas capacidades cognitivas. Ello no implica necesariamente una repercusión significativa en la funcionalidad de la persona, más allá de la propia del devenir de los años, pero en algunas ocasiones esta repercusión puede darse.

El concepto de *deterioro cognitivo*, introducido por Petersen en 1999^1^, hace referencia a una condición patológica en la que la función cognitiva se encuentra por debajo del rendimiento normal esperable por edad y, en una proporción elevada de casos, es un estado transicional entre una cognición conservada y la demencia^2^.

Para diagnosticar deterioro cognitivo es preciso detectar mediante pruebas estandarizadas medibles dos aspectos: primero, que el rendimiento en algún área (memoria, atención, lenguaje y/o razonamiento) está por debajo del valor de normalidad, y segundo, que -a diferencia de la demencia- dicho rendimiento no interfiere significativamente en el funcionamiento diario de la persona en cuanto a su autonomía personal. Existen numerosas pruebas que pueden aplicarse para este despistaje, pero es fundamental que los valores normales estén estandarizados en la población diana en la que vayan a utilizarse^3^, a fin de poder diferenciar el deterioro cognitivo del cambio cognitivo o las quejas subjetivas, en las que no se demuestra un peor rendimiento comparado con el grupo etario, y ajustado por nivel educativo.

El deterioro cognitivo asociado a la edad es un tema de enorme relevancia, debido al aumento de personas de edad avanzada en los diferentes países, incluido el nuestro. El envejecimiento de la población mundial ha multiplicado la prevalencia del deterioro cognitivo (estimado entre un 6,7 y un 25,2 % en adultos mayores de 65 años)^4^ y la cifra aumenta con la edad y el menor nivel educativo, especialmente en varones^4^. Estas cifras son variables en función del país, el tipo y diseño del estudio, y los criterios de diagnóstico de deterioro cognitivo^5^. No obstante, es evidente el enorme impacto que el deterioro cognitivo y la demencia tienen a nivel público, en términos de pérdida de esperanza de vida activa y aumento de dependencia, con la repercusión sanitaria, social y económica que ello conlleva.

La causa más común del deterioro cognitivo son las enfermedades neurodegenerativas. Se estima que un 10-15 % de las personas con deterioro cognitivo evolucionan anualmente a demencia^6^. En algunos casos, esta evolución puede obedecer a causas tratables -siendo, por tanto, potencialmente reversibles- y en otros, al menos permanecer estables en su evolución.

Los factores más relevantes asociados al deterioro cognitivo entre la población geriátrica son la edad, el bajo nivel educativo y la escasa reserva cognitiva, la depresión, los factores de riesgo cardiovascular, el estrato socioeconómico, y el estado de actividad física. Como podemos observar, estos factores están influidos de modo directo por el entorno y sus condiciones, y (documento en elaboración)los cambios ambientales que se produzcan. Muy recientemente, tenemos el caso de la pandemia de COVID-19, donde se ha observado que el confinamiento derivado de ella intensificó el deterioro cognitivo y funcional, especialmente en aquellas personas con alteraciones cognitivas previas, como han descrito Isabel San Martín-Erice y colaboradores en su artículo *High-risk profiles for functional and cognitive decline following COVID-19 confinement in nursing home residents: A multicentre observational study*^7^, publicado en este número 49(2) de la revista Anales del Sistema Sanitario de Navarra.

Es clave identificar estos factores lo más precozmente posible, pues suponen una ventana de oportunidad para implementar intervenciones encaminadas a ralentizar la progresión de los síntomas deficitarios y retrasar, por tanto, la conversión a demencia y sus efectos negativos, como son la pérdida de autonomía y el aumento de dependencia. En este contexto, y en el marco del Plan de Atención a la Demencia del Servicio Navarro de Salud-Osasunbidea, entre otras acciones, se ha revisado y actualizado la Guía de actuación en el abordaje de la demencia Atención Primaria-Neurología (documento en elaboración). En ella se incide en la detección precoz del deterioro cognitivo utilizando, además de la historia clínica, tests de cribado más sensibles. Además, la incorporación de biomarcadores en la práctica clínica habitual posibilita el diagnóstico de la enfermedad de Alzheimer en etapas más tempranas. Todo ello debe contribuir a promover e implementar medidas para fomentar la salud cerebral y retrasar, en la medida de lo posible, la progresión del deterioro cognitivo.

Junto con las intervenciones farmacológicas, desde hace décadas se vienen desarrollando otras terapias no farmacológicas, sobre las que cada vez se dispone de más evidencia favorable^8^. Bajo el término terapias no farmacológicas se agrupan múltiples y diversas intervenciones como son la estimulación, entrenamiento y rehabilitación cognitivas, el entrenamiento en actividades de vida diaria (AVD), las intervenciones sensoriales, la musicoterapia, o la terapia con animales, entre otras. Estas intervenciones proponen programas terapéuticos estructurados por niveles según capacidad cognitiva (de modo individualizado) y en diferentes modalidades (formato grupal y/o individual, de tipo presencial o vía *on-line*), implementados por equipos multidisciplinares, y donde se incluye el asesoramiento a familiares.

Actualmente, existe unanimidad en considerar estas terapias como una parte importante del tratamiento, y se recomienda su utilización, ya que son muchos los estudios que vienen respaldando la eficacia de las intervenciones no farmacológicas en población con deterioro cognitivo en distinto grado, observándose una mejoría cognitiva global y de otras variables, como el estado de ánimo depresivo^9^. En este mismo número de la revista, Isabel Gómez-Soria y colaboradores describen cómo los pacientes con deterioro cognitivo leve sometidos a un programa de estimulación cognitiva^10^ mostraron, a los 12 meses de seguimiento, puntuaciones de ansiedad y depresión significativamente mejores respecto del inicio, mientras que el grupo control empeoró^11^.

Para minimizar el impacto de esta condición por el coste que representa para el individuo, las familias y los sistemas sanitarios, es crucial que las autoridades sanitarias elaboren y desarrollen planes de salud capaces de prevenir, identificar y desarrollar actuaciones. Es fundamental la coordinación y trabajo en red de los diferentes agentes de Salud (Atención Primaria, Neurología, Geriatría, Salud Mental...) y otras instancias como Derechos Sociales, entre otros, así como la incorporación de los pacientes y familiares, para tratar de garantizar un abordaje integral de este proceso.

Estas medidas deben plantearse desde un marco de la promoción de un envejecimiento saludable, que prevenga la enfermedad y que reduzca la duración de la discapacidad, si esta se da. Este envejecimiento saludable será el resultado de la promoción de la actividad física y mental, la participación social, y la atención a nuestra salud emocional. Como dice una máxima del psicólogo canadiense Donald Hebb referida a nuestras capacidades cognitivas, popularizada y adaptada, *Use it or lose it* (úsala o la perderás)^12^.
